# Modulation of Kinase Activities In Vitro by Hepatitis C Virus Protease NS3/NS4A Mediated-Cleavage of Key Immune Modulator Kinases

**DOI:** 10.3390/cells12030406

**Published:** 2023-01-25

**Authors:** Mohd Amir F. Abdullah, Sarah M. McWhirter, Zucai Suo

**Affiliations:** 1 Department of Biochemistry, The Ohio State University, Columbus, OH 43210, USA; 2 Department of Molecular and Cellular Biology, Harvard University, Cambridge, MA 02138, USA; 3 Department of Biomedical Sciences, Florida State University College of Medicine, Tallahassee, FL 32306, USA

**Keywords:** IKKα, IKKβ, IKKε, TBK1, HCV protease, kinase regulatory domain

## Abstract

Hepatitis C Virus NS3/NS4A, a serine protease complex, has been found to interact with many host proteins and cause various adverse effects on cellular function and immune response. For example, the cleavage of important immune factors by NS3/NS4A has been suggested as a mechanism for the hepatitis C virus to evade innate immunity. The spectrum of susceptible substrates for NS3/NS4A cleavage certainly includes important immune modulator kinases such as IKKα, IKKβ, IKKε, and TBK1, as demonstrated in this paper. We show that the kinase activities of these four host kinases were transformed in unexpected ways by NS3/NS4A. Treatment with NS3/NS4A caused a significant reduction in the kinase activities of both IKKα and IKKβ, suggesting that HCV might use its NS3/NS4A protease activity to deactivate the NF-κB-associated innate immune responses. In contrast, the kinase activities of both IKKε and TBK1 were enhanced after NS3/NS4A treatment, and more strikingly, the enhancement was more than 10-fold within 20 min of treatment. Our mass spectroscopic results suggested that the cleavage after Cys89 in the kinase domain of IKKε by NS3/NS4A led to their higher kinase activities, and three potential mechanisms were discussed. The observed kinase activity enhancement might facilitate the activation of both IKKε- and TBK1-dependent cellular antiviral pathways, likely contributing to spontaneous clearance of the virus and observed acute HCV infection. After longer than 20 min cleavage, both IKKε- and TBK1 gradually lost their kinase activities and the relevant antiviral pathways were expected to be inactivated, facilitating the establishment of chronic HCV infection.

## 1. Introduction

Liver diseases caused by the hepatitis C virus (HCV) are a serious health problem. About 30% of HCV infection is acute infection since infected people can clear the virus within 6 months of infection without any treatment. The remaining 70% of HCV infections are chronic infections that may persist for decades. It is estimated that 10% to 20% of chronically infected people will develop liver cirrhosis, while 1% to 5% will develop hepatocellular carcinoma [1]. Currently, an estimated 58 million people worldwide have chronic HCV infection, with about 1.5 million new infections occurring per year. Due to the availability of cost-effective and efficacious generic direct-acting antiviral agents (DAAs), the World Health Organization (WHO) has set up an ambitious target of HCV elimination by 2030 and many countries are working towards the target [2,3,4].

The HCV genome is a plus-stranded RNA about 10 kb in length, and its organization is similar to that of members of the family Flaviviridae [5,6,7]. Based on sequence analysis, seven major HCV genotypes have been identified and each of them has sub-genotypes [8]. The genome encodes a single precursor protein that includes structural and nonstructural proteins [6,9]. The precursor protein is proteolytically processed by both host signal peptidases and viral proteases to produce at least 10 viral proteins: Core, E1, E2, p7, NS2, NS3, NS4A, NS4B, NS5A, and NS5B [9,10,11]. For example, the NS4A/NS4B, NS4B/NS5A, and NS5A/NS5B junctions are cleaved in trans by a serine protease complex, NS3/NS4A [12] while the NS3/NS4A junction is cleaved in cis by NS3 [13]. The NS3 protein is a multidomain protein of about 70 kDa. The amino-terminal third of NS3 forms a chymotrypsin-like serine protease domain [12,14,15] while the carboxyl-terminal two-thirds possesses both ATPase and RNA helicase activities [16,17]. Based on the analysis of 1568 HCV NS3 sequences derived from patients infected with HCV (genotypes 1 to 6), 85 of 181 amino acid residues of the NS3 protease domain possess less than 1% variability [18]. The consensus sequence alignment demonstrates that the catalytic triad residues His57, Asp81, and Ser139 for the NS3 protease [19] are conserved across all HCV genotypes. NS4A, a 7-kDa and 54 amino acid residue protein, acts as a cofactor of the NS3 protease. The N-terminal 20 residues of NS4A are predicted to form a hydrophobic trans-membrane α-helix [8,14], which presumably inserts into the ER membrane to anchor the HCV replicase [8]. The NS4A residues 22–31 (consensus sequence: SVVIVGRIIL) constitute the hydrophobic core, which interacts with two β-strands of the NS3 protease domain [7,8,9,10,11,12,13,19]. In the absence of NS4A, the NS3 protease activity decreases significantly, e.g., a ~950-fold decrease at the cleavage of the NS4A/NS4B junction [11]. After several decades of development of direct-acting anti-HCV drugs, quite a few small molecule inhibitors, targeting NS3/NS4A protease, NS5B RNA polymerase, and NS5A, have been approved by the Food and Drug Administration since 2011 and their various combinations have been widely used as therapies to treat HCV infection with good tolerability and greater than 90% sustained virological response [20,21].

Intracellularly, NS3 has been shown to bind to the catalytic subunit of protein kinase A (PKA) which prevents the nuclear translocation of this subunit and affects many PKA functions [22]. The activation of oncogene products via cleavage by the NS3/NS4A protease has been suggested as a possible role in the development of hepatocellular carcinoma by HCV [23]. NS3 is also shown to have oncogenic activity in NIH 3T3 mouse fibroblast cells [23], rat fibroblast cells [24], and human hepatocyte lines [25]. Additionally, NS3 and HCV core have been found to activate inflammatory pathways in monocytes via toll-like receptors [26].

Interestingly, the protease activity of NS3/NS4A has been implicated in the inhibition of cellular antiviral pathways, leading to persistent HCV infections. Foy et al. first demonstrated that the NS3/4A protease blocks the phosphorylation and effector action of interferon regulatory factor-3 (IRF-3) [27]. They subsequently found that NS3/NS4A depressed retinoic acid-inducible gene I (RIG-I) signaling [28]. Meanwhile, Li et al. discovered that NS3/NS4A cleaves toll-like receptor 3 (TLR3) adaptor protein, TRIF [29]. Moreover, two other groups discovered that NS3/4A cleaves an adaptor protein in the RIG-I antiviral pathway, resulting in the shutting down of RIG-I-dependent antiviral responses [30,31].

In addition to these signaling molecules, we hypothesized that NS3/NS4A may cleave other proteins involved in innate immunity and antiviral pathways, e.g., cellular kinases such as Inhibitor of κB kinase α (IKKα), IKKβ, IKKε, and TBK1. These kinases are known to phosphorylate IκB proteins to activate transcription factors nuclear factor-κB (NF-κB), IRF-3, and IRF-7 [32,33], which are central to cellular immune and inflammatory responses and cell survival in higher eukaryotes [34]. Specifically, the canonical NF-κB signaling pathway is mediated by IKKβ while the non-canonical pathway depends on IKKα [35]. IKKβ is primarily responsible for the phosphorylation of specific residues of prototypical IκB proteins (IκBα, IκBβ, and IκBε) bound to NF-κB as well as an atypical IκB protein, p105, which is the precursor of the NF-κB subunit p50 [36]. IKKα phosphorylates specific residues of p100 to convert it to the NF-κB subunit p52 [35]. IKKε and TBK1 directly phosphorylate IRF3 and IRF7 and such phosphorylation promotes the dimerization and nuclear translocation of these transcription factors that stimulate the production of type I interferons [37]. IKKε and TBK1 have also been found to play a role in oncogenic transformation and thereby become novel drug targets with applications in the treatment of cancer and a variety of inflammatory diseases including rheumatoid arthritis and obesity-related metabolic disorders [38]. To confirm our aforementioned hypothesis, we performed in vitro cleavage of these kinases by recombinant NS3/NS4A. Our results demonstrate that NS3/NS4A can cleave and regulate the kinase activities of IKKα, IKKβ, IKKε, and TBK1.

## 2. Experimental Procedures

### 2.1. Peptide Synthesis

All peptides utilized in this study were chemically synthesized and HPLC purified by Peptides International Inc. (Louisville, KY, USA). All synthetic peptides were confirmed to be the desired ones by analyses of amino acid composition and electron spray mass spectrometry. Their purities exceeded 95%, and they were stored frozen at −80 °C as 10 mM stock solutions in deionized water or 100% dimethyl sulfoxide.

### 2.2. Protein Expression and Purification of HCV NS3

**Figure 1:**
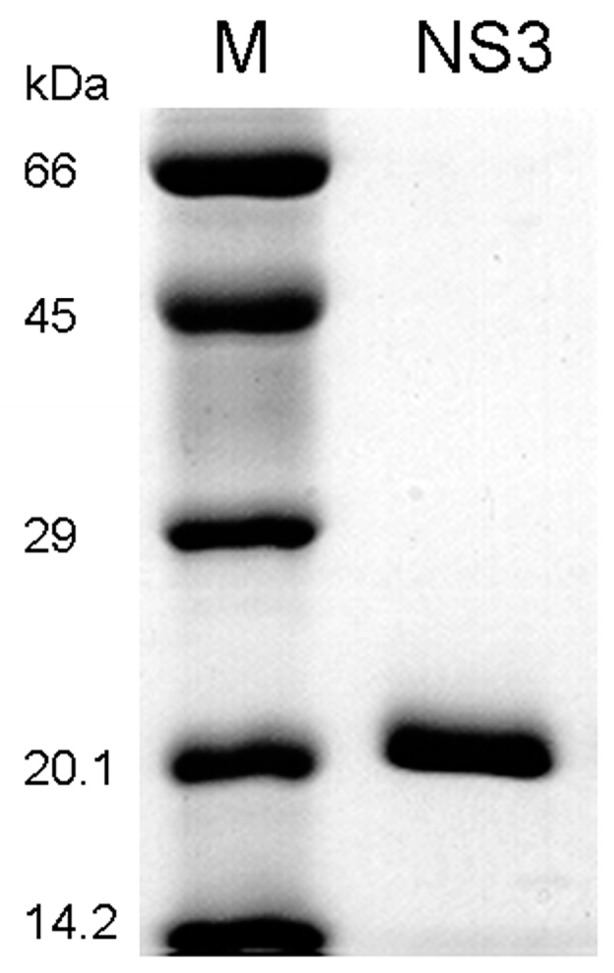
SDS-PAGE of purified HCV NS3 protease domain expressed in *E. coli*. The NS3 was purified through a Ni-NTA affinity column, followed by a heparin sepharose column, and finally a MonoS 10/10 cation exchange column. The left lane is protein size markers, and the right lane is purified NS3 (20 kDa).

The N-terminal domain (residues 1–182) of the NS3 protein is as efficient as the full-length NS3 protein in proteolytic activity [39]. We have cloned the N-terminal protease domain and expressed the NS3 fragment in *Escherichia coli* BL21 (DE3) Gold (MilliporeSigma, Burlington, MA, USA). The truncated NS3 protein also contains a C-terminus His_6_ tag to facilitate the purification process. The cells were induced with 0.4 mM IPTG at room temperature to increase the solubility of NS3 in the cytoplasmic extract [40]. The NS3 was purified through a Ni-NTA affinity column, a heparin sepharose column, and finally a Mono S 10/10 cation exchange column [40,41,42,43]. After concentration, the protein was flash-frozen with liquid nitrogen before storage at −80 °C. SDS-PAGE of the purified NS3 showed a single protein band at 20.1 kDa (Figure 1), indicating that the protein was highly purified. The concentration of the purified NS3 protease domain was measured spectrophotometrically at 280 nm using the calculated molar extinction coefficient of 17,990 M^−1^cm^−1^.

### 2.3. Purification of Recombinant Human IKKα, IKKβ, IKKε, and TBK 1

IKKα, IKKβ, and IKKε were purified as described previously [44,45]. Flag-TBK1 was cloned into pFastBac (Life Technologies, Carlsbad, CA, USA). Recombinant bacmids and baculovirus were prepared according to the manufacturer’s instructions. Flag-TBK1 was isolated from baculovirus-infected Sf9 cells and purified using M2-agarose Affinity Gel (SigmaAldrich, St. Louis, MO, USA) equilibrated with 50 mM Tris, pH 8.0, 250 mM NaCl, 0.5 mM NaF, 0.5 mM DTT, 0.1% Tween-20, and 10% Glycerol, and eluted with 0.34 mg/mL flag peptide (Sigma) in the same buffer. The concentrations of the kinases were determined using the Bradford assay (Bio-Rad, Hercules, CA, USA) with BSA as a standard.

### 2.4. Kinase Digestion Condition for Western Blot Analysis

Purified NS3 (6.4 μM) was preincubated with 94 μM of the NS4A synthetic peptide (KKKGSVVIVGRIILS) from Peptides International Inc. on ice for 40 min in the 1X digestion buffer (50 mM HEPES, pH 7.8, 0.5 mM EDTA, 25% glycerol, 10 mM DTT, 0.25 M KCl, 0.05 M NaCl, 0.2% CHAPS) to allow maximum interaction between NS3 and its cofactor NS4A peptide. The affinity of the NS4A peptide to NS3 is lower in 20% glycerol-containing buffers than in a solution containing 50% glycerol [46]. Water and kinases (1.2 μg) were added to a final reaction volume of 25 μL. The reaction was incubated at 23 °C for 29 h. The reactions were stopped by adding SDS loading dye and heated at 95 °C for 3 min and electrophoresed on an 8% SDS-PAGE gel. The separated proteins were transferred to a PVDF membrane, and immunoblotted with the following antibodies: anti-IKKα (sc7606, Santa Cruz Biotechnology, Dallas, TX, USA), anti-IKKβ (IMG-159, Imgenex Corp., San Diego, CA, USA), anti-TBK1 (IMG-139), and anti-IKKε [45].

### 2.5. MALDI-TOF and Nano-LC MS/MS Analysis

Purified NS3 (6.4 μM) was preincubated with 134 μM NS4A synthetic peptide on ice for 40 min in the 1X digestion buffer to allow maximum interaction between NS3 and its cofactor NS4A peptide. IKKε (8 μg)and deionized water were added to a final reaction volume of 25 μL. The reaction was incubated at 23 °C for 30 min. The reaction was stopped by adding SDS loading dye and heated at 95 °C for 3 min and electrophoresed on an 8% SDS-PAGE gel. The ~70-kDa cleavage product of IKKε separated on the SDS-PAGE gel was in gel digested with trypsin and analyzed by Matrix-Assisted Laser Desorption/Ionization Time-Of-Flight mass spectrometry (MALDI-TOF) and Capillary-liquid chromatography-tandem mass spectrometry (Nano-LC MS/MS) at the CCIC Mass Spectrometry and Proteomics Facility of The Ohio State University.

### 2.6. Kinase Digestion Conditions for Pre-Steady-State Kinetic Assays

To achieve maximal interaction between NS3 and the NS4A peptide, high concentrations of the NS4A peptide were used. Previously, the binding affinity of NS3 to NS4A was estimated at about 20 μM [47]. Based on this result, we estimated that about 92% of NS3 will form a protease complex with NS4A if we use 5 μM NS3 and 244 μM NS4A. We performed the single turnover kinetic assay using the same buffer condition as in the digestion above. Digestions were performed on IKKε at 0.1 and 0.5 μM concentrations, which are 50- and 10-fold less than the NS3/NS4A concentration. At different digestion durations, a small aliquot was removed from the reaction and added to an excess amount of gel loading buffer and frozen on a dry ice/methanol bath to stop the reaction. The reaction was electrophoresed on a 5% SDS-PAGE gel, transferred onto a PVDF membrane, and the remaining IKKε substrate was detected using anti-IKKε antibodies. The remaining IKKε concentration was plotted versus time to obtain an exponential decay curve. The curve was fitted to a single exponential equation:

[kinase] = c + a*exp(−*k_obs_*t) (1)

The observed cleavage rate constant (*k_obs_*) and a constant c were yielded. The equilibrium dissociation constant (*K_d_*) of the binding between a kinase and NS3/NS4A was estimated based on the following equation:

*K_d_* = c*(E_t_ − c)/(S_0_ − c) (2)

where S_0_ is the initial kinase concentration, E_t_ is the total concentration of the NS3/NS4A complex, and c is assumed to be the concentration of unbound kinase.

### 2.7. Kinase Digestion Condition for Kinase Activity Assay

Purified NS3 (1.25 μM) was preincubated with the NS4A peptide (325 μM) on ice for 15 min followed by 15 min at 23 °C in the 5X concentrated digestion buffer. A kinase (0.5 μg) was added to a final reaction volume of 50 μL and incubated at 23 °C. Aliquots (5 μL each) were removed from the reaction at 1, 4, 8, 16, 32, 60, 300, 900, 1200 min and flash-frozen in a dry ice/methanol bath and kept in storage at −80 °C until use. A control reaction without NS3/NS4A was also performed to test the stability of the kinases in the digestion buffer for the duration of the digestion.

### 2.8. Protease Inhibition Assay

Pefabloc SC (final concentration 2 mg/mL, Roche, Basel, Switzerland), was added at the beginning of the 15 min preincubation of NS3 and NS4A at 23 °C (before the addition of IKKε) or after the reaction was completed (prior to the flash-freezing step). Four reaction conditions were set up: (i) IKKε without NS3/NS4A; (ii) IKKε with NS3/NS4A; (iii) IKKε with NS3/NS4A pre-treated with Pefabloc; and (iv) IKKε with NS3/NS4A (Pefabloc was added after 16 min of digestion). The reactions were performed at 23 °C for 16 min, and stopped by flash-freezing in a dry ice/methanol bath. The kinase activity of the treated IKKε was measured using GST-IκBα (residues 5–55) as substrate.

### 2.9. Kinase Activity Assay

Kinase activity assays were performed by incubating 1.5 μL of the digested kinases with a 10 μL cocktail (20 mM HEPES, pH 7.6, 50 mM NaCl, 20 mM beta-glycerol phosphate, 1 mM sodium vanadate, 10 mM MgCl_2_, 1 mM DTT, 0.1 mg/mL substrate (GST-IκBα (residues 5–55), GST-IκBα mutant S32A/S36A), 0.1 mM ATP, 0.015 μCi/μL [γ-^32^P] ATP) at 23 °C for 30 min. The reactions were stopped by adding SDS loading dye. The samples were separated by SDS-PAGE, and the gel was stained with Coomassie blue staining solution. Band intensities were determined to ensure equal sample loading. The gel was dried and then quantitated with a PhosphorImager 445 SI (Molecular Dynamics). Radioactive intensities were determined from the bands (normalized to the amount of substrate detected in the gel from the Coomassie stain) to measure kinase activity. Relative kinase activity was determined by calculating the ratio of the kinase activity of NS3/NS4A-treated kinase over the control kinase (in digestion buffer without NS3/NS4A) from the same digestion duration.

## 3. Results

### 3.1. IKKα, IKKβ, IKKε, and TBK1 Are Substrates for NS3/NS4A Protease In Vitro

The consensus sequence of (D/E)XXXXC(A/S) (X, any amino acid residue; the scissile bond, between Cys and Ala or Ser) has been determined for all trans cleavage sites (Table 1) in viral processing of the HCV polyprotein precursor by NS3/NS4A [9,48]. Interestingly, protein sequence alignments in Table 1 suggest that IKKα, IKKβ, IKKε, and TBK1 all contain potential NS3/NS4A cleavage sites which are similar in sequence to those junctions in the HCV polyprotein precursor. To examine whether NS3/NS4A can cleave these kinases, a recombinant NS3 protease domain (residues 1–182) containing a C-terminal hexahistidine tag was chromatographically purified from *E. coli* with at least 95% purity (Figure 1). Since the NS4A residues 22–31 (SVVIVGRIIL) constitute the hydrophobic core, which binds to and activates NS3 [7,8,9,10,11,12,13], a synthetic peptide with the sequence of KKGSVVIVGRIILSGK (the underlined residues are from the consensus HCV NS4A sequence while the three Lys residues were added to increase the aqueous solubility of the peptide) was used as the NS4A cofactor in all in vitro digestion assays in this paper. To examine the protease activity of the NS3/NS4A complex, an internally quenched fluorogenic substrate (sequence: Ac-Asp-Glu-Asp(Edans)-Glu-Glu-Abu ψ[COO]-Ala-Ser-Lys(Dabcyl)-NH_2_; Abu, 2-aminobutyric acid; Ac, acetylation; Dabcyl, 4-[[4′-(dimethylamino)phenyl]azo]benzoic acid; Edans, 5-[(2′-aminoethyl)amino]naphthalenesulfonic acid; ψ[COO], the ester bond between Abu and *L*-(+)-lactic acid) [49] was used as the substrate. After cleavage of the peptide bond Abu ψ[COO]-Ala by NS3/NS4A, the fluorescence of the donor (Edans) increased. The steady-state protease activity (355,000 M^−1^s^−1^) of the NS3/NS4A complex was close to 345,000 M^−1^s^−1^ measured by Taliani et al. [49], indicating that our assembled NS3/NS4A complex was active. To investigate if the steady-state protease activity was affected by the molar ratio of NS3/NS4A, 2 μM of the internally quenched fluorogenic peptide substrate was added to a preincubated solution of 20 nM NS3 and the NS4A peptide with a concentration varied from 800 nM to 6 µM for 0 to 3 min at 23 °C. The steady-state protease activity measured based on the time-dependent donor fluorescence change was comparable, suggesting that the substrate cleavage kinetics was not significantly influenced by the change of the molar ratio of NS3/NS4A from 40- to 300-fold.

**Figure 2:**
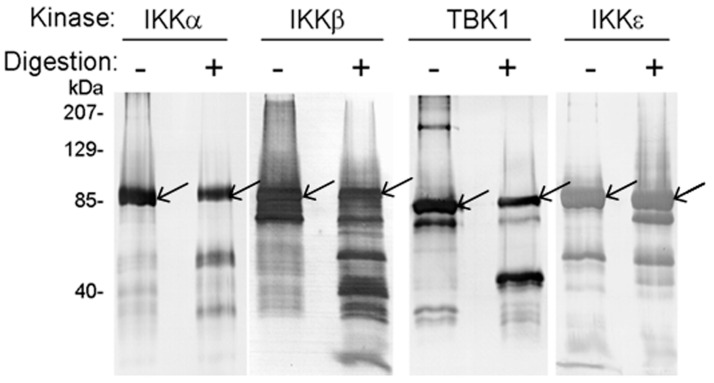
Western blot analysis of in vitro digestion of recombinant human IKKα, IKKβ, IKKε, and TBK1 with NS3/NS4A. The kinases (1.2 μg) were digested with NS3/NS4A (6.4 μM NS3 + 94 μM NS4A) at 23 °C for 29 h. Arrows indicate bands of intact kinases.

The four human kinases were expressed and purified from baculovirus-infected insect cells (Experimental Procedures). Figure 2 shows that IKKα, IKKβ, IKKε, and TBK1 proteins were susceptible to degradation catalyzed by NS3/NS4A. To our knowledge, this is the first report of NS3/NS4A having a direct proteolytic effect on these human kinases. Notably, IKKα and IKKβ were cleaved into several small fragments after 29 h at 23 °C while one large cleavage product was dominant in the degradation reactions of IKKε and TBK1, e.g., the ~70-kDa cleavage product of IKKε (the band immediately below the intact IKKε protein band). As a negative control reaction, Dpo4, a 40-kDa Y-Family DNA polymerase from *Sulfolobus solfataricus* [50,51,52] that does not contain the NS3/NS4A cleavage sites (Table 1) was not cleaved by NS3/NS4A. This indicates that NS3/NS4A did not randomly cleave proteins in our reaction conditions. When the ~70-kDa cleavage product of IKKε was sequenced through in gel trypsin digestion and then mass spectrometric analysis (MALDI-TOF and Nano-LC MS/MS, see Experimental Procedures), we discovered that peptide coverage was from residue 118 (cleavage after Arg117 by trypsin) to the C-terminus of IKKε. This suggested that the two predicted NS3/NS4A cleavage sites (after Cys509, and Cys626) in IKKε (Table 1) were inaccessible to the protease during degradation. Cleavage after Cys89 (the third predicted NS3/NS4A cleavage site in IKKε) would produce a large ~70-kDa peptide, corresponding to the cleavage product observed in Figure 2, and a small ~15-kDa peptide that was not observed in Figure 2. The reason why residues 90–117 were not identified by our mass spectrometric analysis was not clear. Similarly, cleavage of TBK1 at the predicted NS3/NS4A cleavage site (after Cys267, Table 1) would produce a large ~50-kDa peptide (corresponding to the cleavage product observed in Figure 2) and a smaller ~30-kDa peptide (not observed in Figure 2). The generation of the ~50-kDa peptide was confirmed through MALDI-TOF and Nano-LC MS/MS analysis of the large TBK1 cleavage product, which covered residue 268 to the C-terminus of TBK1.

### 3.2. Estimation of the Substrate Specificity of IKKε by Pre-Steady-State Kinetic Methods

**Figure 3:**
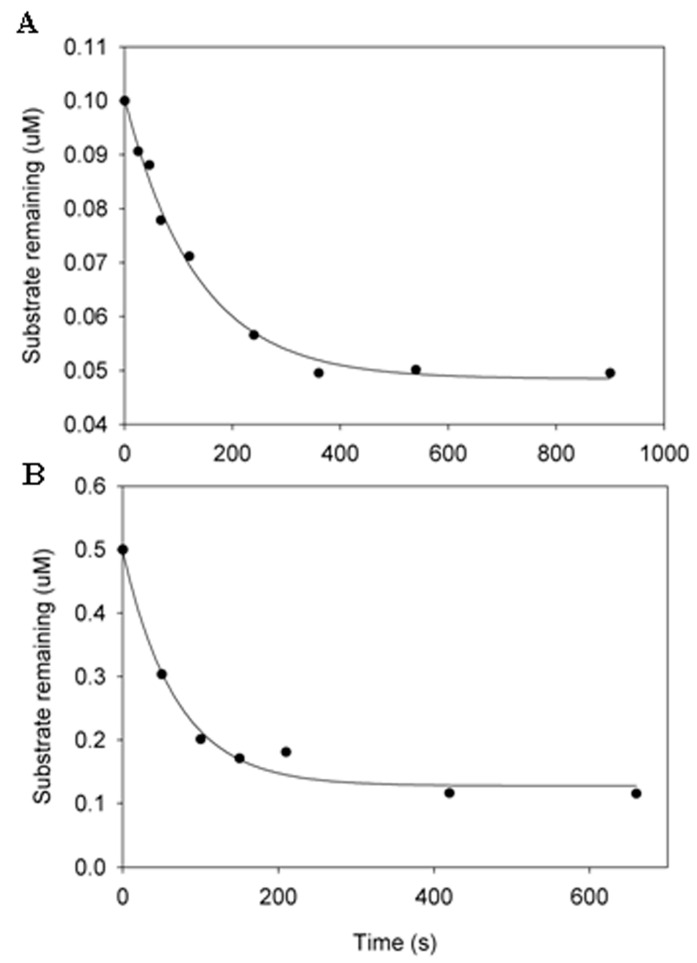
Pre-steady-state kinetic analysis of the cleavage of IKKε by NS3/NS4A (5 μM NS3 + 244 μM NS4A) at 23 °C. The IKKε concentration was 0.1 μM (**A**) and 0.5 μM (**B**). In each time course, the concentration of remaining IKKε was plotted against time. The data were fit to Equation (1) (see Experimental Procedures) to yield 0.0075 and 0.0145 s^−1^ for the observed cleavage rate constants in (**A**,**B**), respectively.

To examine if NS3/NS4A cleaves these four kinases with reasonable efficiency and to avoid the complication of product inhibition, we measured the cleavage efficiency of IKKε at 23 °C under single-turnover conditions. At least a 10-fold excess of NS3/NS4A over IKKε was used in the kinetic assay. As a kinetic strategy, the single-turnover kinetic assay was employed to estimate the cleavage rate of a human kinase by NS3/NS4A. It does not mean that the single-turnover reaction conditions would be certainly encountered in vivo. The single-turnover assay was performed by using 5.0 μM NS3/NS4A with one of two different IKKε concentrations and the data were fit into a single exponential equation, Equation (1) (see Experimental Procedures). The observed cleavage rate constants (*k_obs_*) at IKKε concentrations of 0.1 and 0.5 μM were determined to be 0.0075 and 0.0145 s^−1^, respectively (Figure 3). Due to the lack of enough protein substrate IKKε, we did not determine the substrate concentration dependence of *k_obs_* which can yield the maximum *k_p_* and the equilibrium dissociation constant *K_d_* for the binding of IKKε to NS3/NS4A. However, we can estimate the *K_d_* from these time courses shown in Figure 3. By assuming the constant in Equation (1) to be the concentration of unbound IKKε in a cleavage reaction, we estimated the *K_d_* to be 4.57 and 1.68 μM in the presence of 0.1 and 0.5 μM IKKε, respectively (see Experimental Procedures). These low micromolar *K_d_* values indicate the binding between the protease complex NS3/NS4A and IKKε is moderately strong. The substrate specificity *k_obs_*/*K_d_* for the degradation of 0.1 and 0.5 μM IKKε was estimated to be 1641 and 8631 M^−1^s^−1^, respectively. Previously, steady-state kinetic analysis has been used to estimate the cleavage efficiency (*k_cat_*/*K_m_*) of peptide substrates representing the viral NS4A/NS4B (1600 M^−1^s^−1^) and NS4B/NS5A (110 M^−1^s^−1^) cleavage sites catalyzed by NS3/NS4A at 30 °C [53]. These *k_obs_*/*K_d_* and *k_cat_*/*K_m_* values suggest that IKKε is as good a substrate, if not better, to NS3/NS4A as those HCV junction-mimicking substrates. Thus, HCV NS3/NS4A protease is capable of cleaving IKKε, and possibly IKKα, IKKβ, and TBK1, in vivo.

### 3.3. NS3/NS4A Modulates Kinase Activity

**Figure 4:**
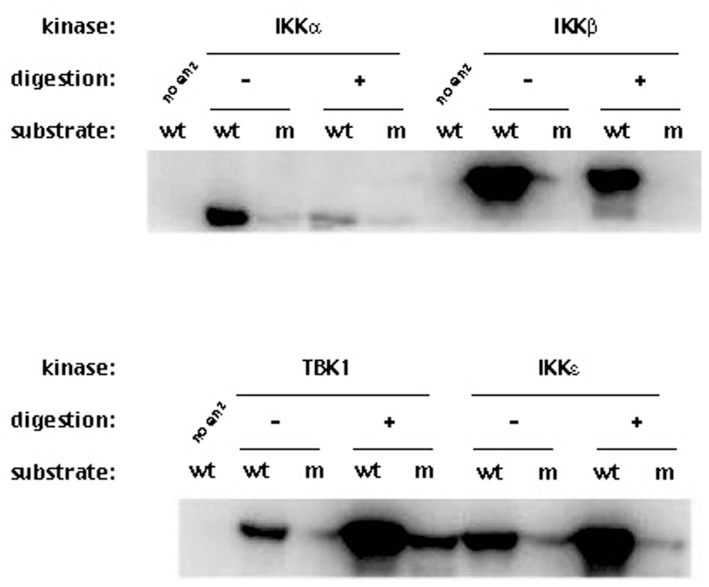
Effect of the proteolytic activity of NS3/NS4A on IKKα, IKKβ, IKKε and TBK1 kinase activities. The kinases (0.5 μg) were treated with NS3/NS4A (1.25 μM NS3 + 325 μM NS4A) at 23 °C for 35 min. The kinase activities were tested using GST-IκBα (residues 5–55) and [γ-^32^P]ATP as substrates. Phosphorylated GST-IκBα bands were detected by exposing to X-ray film. wt, wild type GST-IκBα (residues 5–55); m, GST-IκBα S32A/S36A mutant; no enz, reaction without kinase.

We expected that proteolytic digestion of these four kinases by NS3/NS4A would reduce their respective kinase activities. This assay is more sensitive than the detection of cleavage products since changes in the kinase activity will reflect the physiological changes in the kinases. Since we are only measuring the kinase activity and not the cleavage products, much lower concentrations of NS3 (1.25 μM) and the NS4A peptide (325 μM) were used. Each of those kinases was reacted with NS3/NS4A at 23 °C for 35 min and the reaction was stopped by flash-freezing the reaction in a dry ice/methanol bath. The treated kinases (with or without NS3/NS4A) were then tested for their kinase activities to phosphorylate GST-IκBα (residues 5–55). Figure 4 shows that the kinase activities of IKKα and IKKβ were reduced after treatment with NS3/NS4A, but surprisingly, the kinase activities of IKKε and TBK1 increased after treatment with NS3/NS4A. Moreover, the NS3/NS4A treatment also increased TBK1 phosphorylation of mutant GST-IκBα (residues 5–55) containing Ser32Ala/Ser36Ala, a substrate that was normally not phosphorylated by TBK1, IKKα, IKKβ, and IKKε. This suggests the substrate specificity of TBK1 was altered by NS3/NS4A.

**Figure 5:**
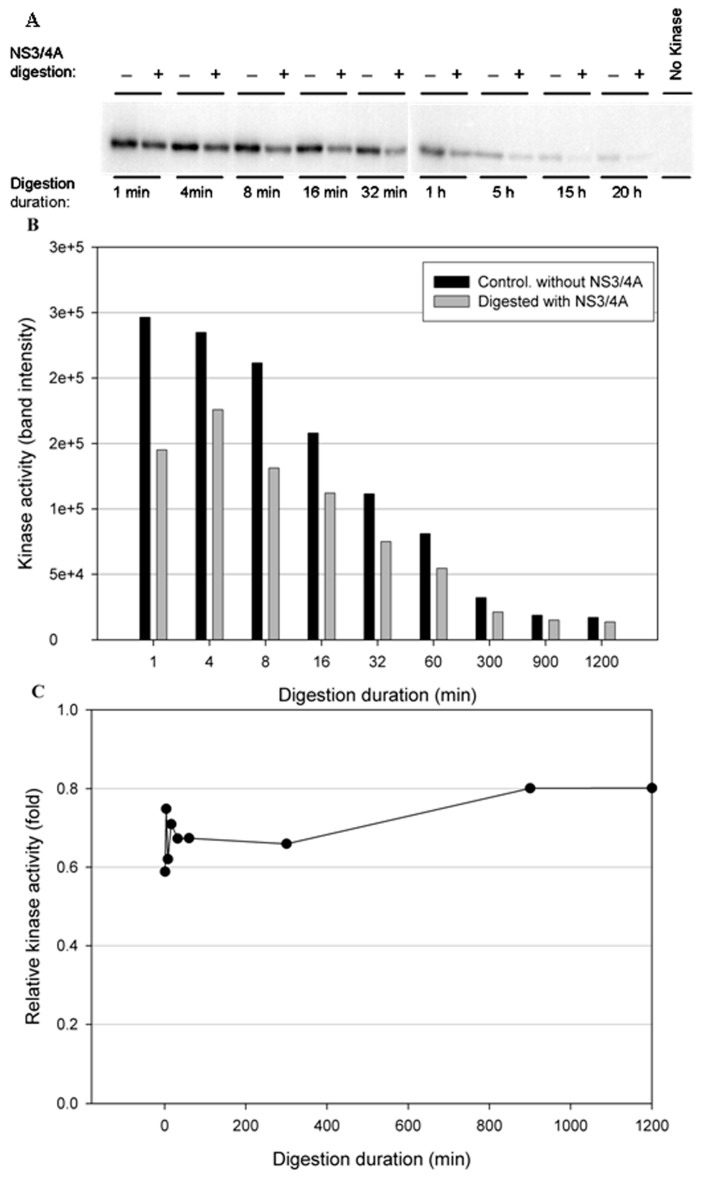
Effect of the proteolytic activity of NS3/NS4A on IKKβ kinase activity in a time-dependent manner. The kinases (0.5 μg) were treated with NS3/NS4A (1.25 μM NS3 + 325 μM NS4A). (**A**) IKKβ was treated with or without NS3/4A for up to 20 h. The kinase activity was tested using GST-IκBα (residues 5–55) and [γ-^32^P]ATP as substrates. Phosphorylated GST-IκBα bands were detected by a PhosphoImager. (**B**) The band intensities detected by the PhosphoImager were plotted versus time. (**C**) The relative kinase activities (IKKβ treated with NS3/NS4A versus untreated control) were plotted versus time.

**Figure 6:**
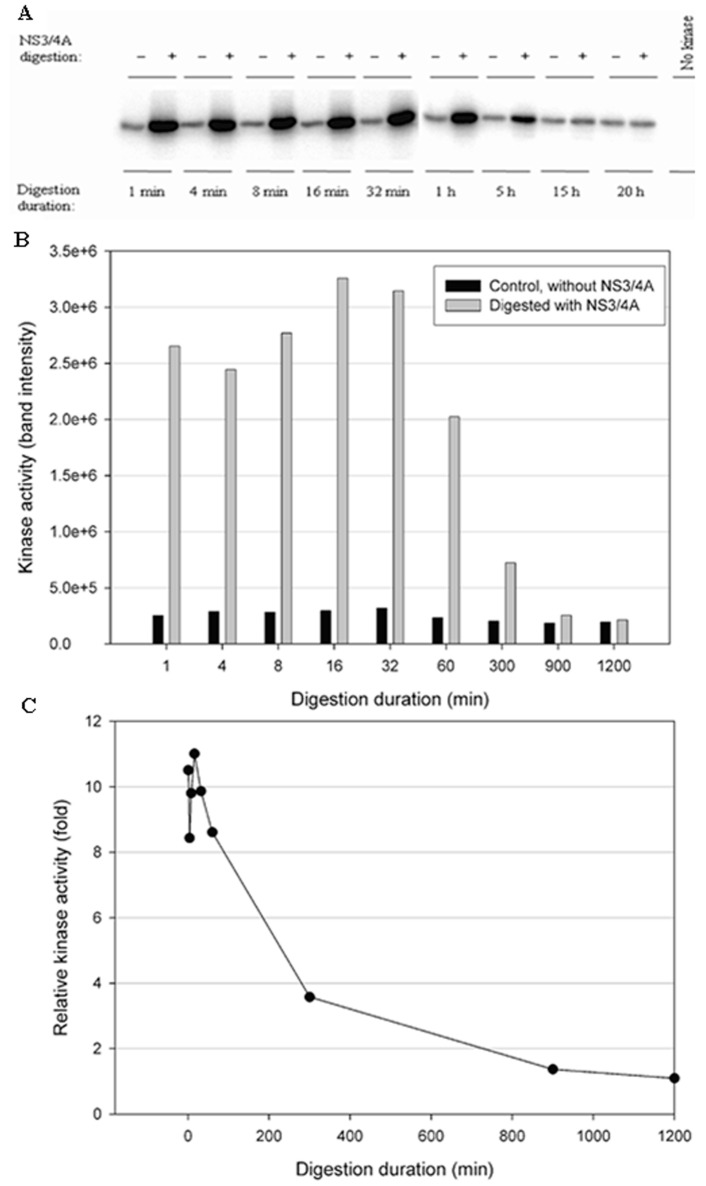
Effect of NS3/NS4A cleavage of IKKε in a time-dependent manner. The kinases (0.5 μg) were treated with NS3/NS4A (1.25 μM NS3 + 325 μM NS4A). (**A**) IKKε was treated with or without NS3/4A up to 20 h. The kinase activity was tested using GST-IκBα (residues 5–55) and [γ-^32^P] ATP as substrates. Phosphorylated GST-IκBα bands were detected by a PhosphoImager. (**B**) The band intensities detected by the PhosphoImager were plotted versus time. (**C**) The relative kinase activities (IKKε treated with NS3/4A versus untreated control) were plotted versus time.

**Figure 7:**
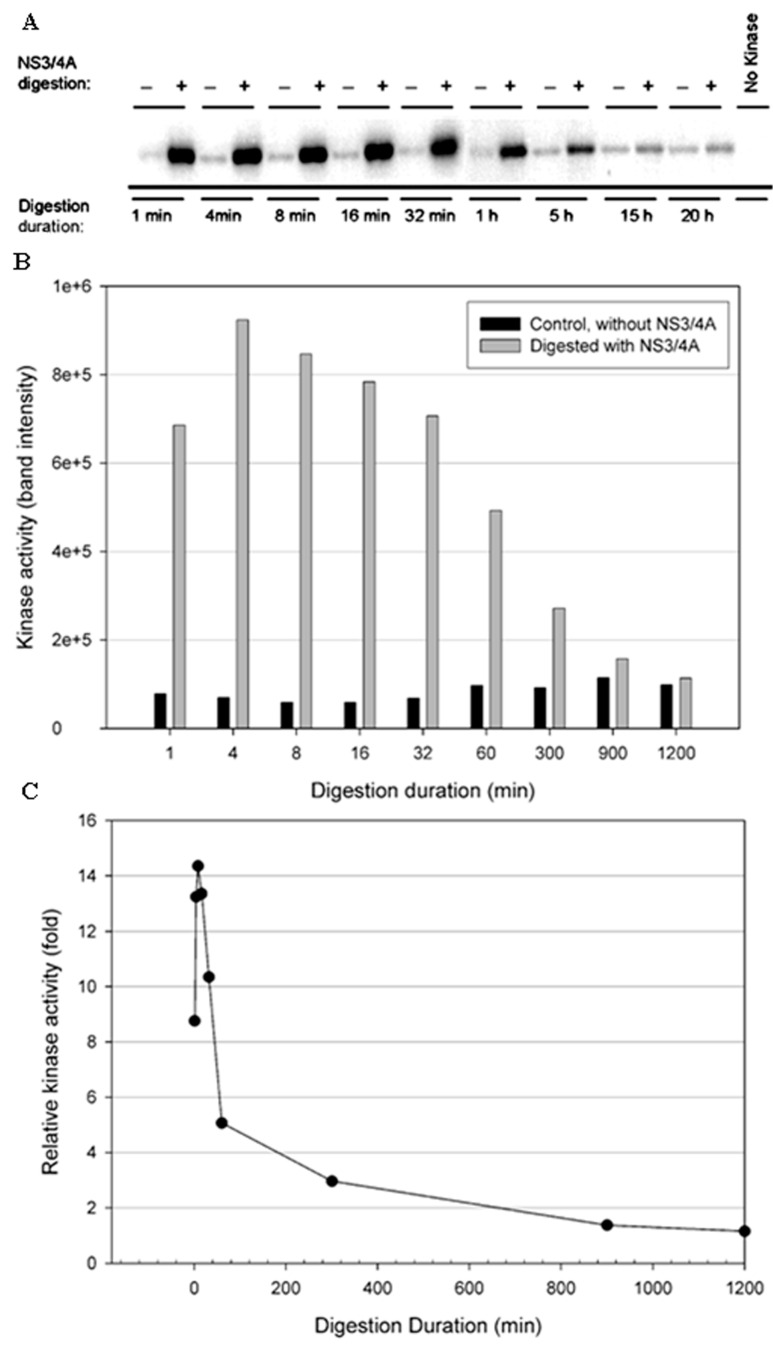
Effect of NS3/NS4A digestion on TBK1 kinase activity in a time-dependent manner. The kinases (0.5 μg) were treated with NS3/NS4A (1.25 μM NS3 + 325 μM NS4A). (**A**) TBK1 was treated with or without NS3/4A up to 20 h. The kinase activity was tested using GST-IκBα (residues 5–55) and [γ-^32^P] ATP as substrates. Phosphorylated GST-IκBα bands were detected by a PhosphoImager. (**B**) The band intensities detected by the PhosphoImager were plotted versus time. (**C**) The relative kinase activities (TBK1 treated with NS3/NS4A versus untreated control) were plotted versus time.

To test the stability of the kinases in the NS3/NS4A digestion buffer condition, a time-course digestion assay was set up at 23 °C. In the control reactions (without NS3/NS4A), the duration of incubation from 1 min to 20 h significantly reduced the kinase activity of IKKβ (Figure 5B), suggesting that IKKβ was not stable. In contrast, the kinase activities of IKKε (Figure 6B) and TBK1 (Figure 7B) were stable over the 20-h incubation, indicating that IKKε and TBK1 were more stable than IKKβ. In Figure 5C, treatment with NS3/NS4A further reduced the kinase activity of IKKβ compared to undigested IKKβ. Amazingly, treatment of IKKε with NS3/NS4A increased kinase activity up to 11-fold within the first 16 min, and then gradually returned to the level of control IKKε over a period of 1200 min (Figure 6C). Similarly, treatment of TBK1 with NS3/NS4A increased kinase activity up to 14-fold within the first 8 min, and then gradually returned to the level of control TBK1 over a period of 1200 min (Figure 7C). The cause of the initial increase in kinase activities of both IKKε and TBK1 by NS3/NS4A may be due to the degradation of a kinase regulatory domain (see Discussion). As these two kinases were further degraded by NS3/NS4A, their overall kinase activities dropped significantly with time. After five hours of cleavage by NS3/NS4A, the remaining kinase activities of digested IKKε and TBK1 were still slightly higher than those of undigested kinases.

### 3.4. The NS3/NS4A Proteolytic Activity Is Essential for Modulation of the Kinase Activities of Both IKKε and TBK1

**Figure 8:**
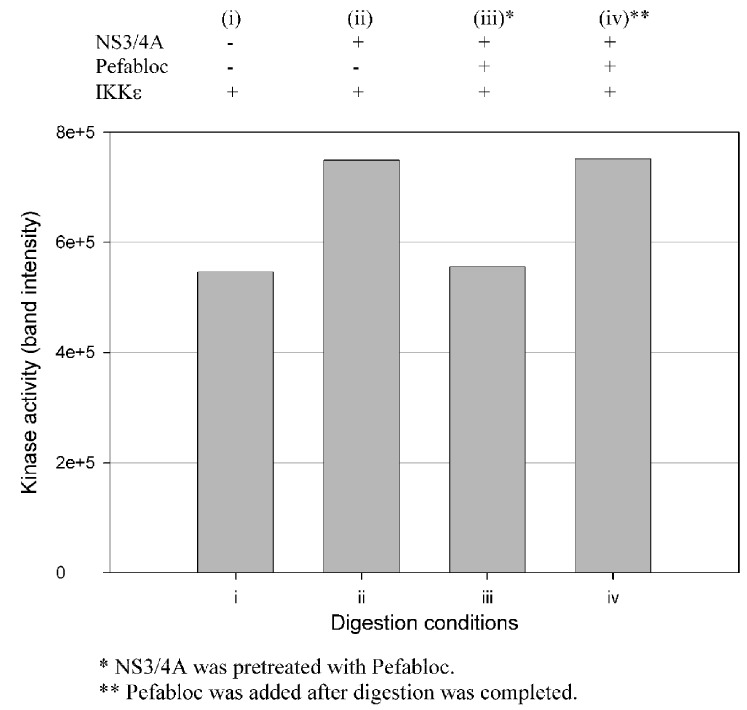
Effect of Pefabloc SC on the capability of NS3/NS4A to enhance the kinase activity of IKKε. Pefabloc SC was added at the beginning of the 15 min preincubation of NS3 and NS4A (1.25 μM NS3 + 325 μM NS4A) at 23 °C (before addition of IKKε) or after the reaction was completed (prior to the flash-freezing step). Four reaction conditions were set up: (i) IKKε not treated with NS3/NS4A; (ii) IKKε treated with NS3/NS4A; (iii) IKKε treated with NS3/NS4A (pre-treated with Pefabloc) and (iv) IKKε treated with NS3/NS4A (Pefabloc added after completion of digestion). The digestions were performed at 23 °C for 16 min, and the reaction was stopped by flash-freezing in dry ice/methanol bath. The kinase activities were measured using GST-IκBα as substrate.

Enhancement of the kinase activities of IKKε and TBK1 during cleavage could be due to either NS3/NS4A mediated cleavage or protein/protein interactions such as the binding of IKKε to NS3/NS4A, or both. To examine these possibilities, we used Pefabloc SC, a broad-spectrum serine protease inhibitor, to covalently modify NS3 and permanently inhibit its serine protease activity. Figure 8 shows that the IKKε kinase activity was not affected by NS3/NS4A which was pretreated with Pefabloc SC, but untreated NS3/NS4A was able to enhance the kinase activity of IKKε. Similar results were also observed with TBK1, suggesting the cleavage of these two kinases by NS3/NS4A was likely the reason for their kinase activity enhancement.

## 4. Discussion

There are many reports about the interactions of HCV proteins with host cell factors involved in cell signaling, apoptosis, transcriptional regulation, transformation, membrane rearrangements, and immuno-modulation, which lead to dysregulation of cellular functions [54]. Among the HCV proteins, NS3 actively interacts with cellular proteins through either direct binding interactions, or its protease activity, to influence cellular processes such as triggering inflammation [26], affecting PKA activity [22], inhibiting cellular antiviral responses [29,55], and cell transformation [23,24,25]. It is likely that more host proteins will be found to interact with NS3 through new assays or methodologies.

In this paper, we used pre-steady-state kinetic methods to examine the cleavage of NS3/NS4A under single turnover reactions, in which the molar excess of the protease complex was reacted with each of the four human kinases (IKKα, IKKβ, IKKε, and TBK1). The single turnover conditions ensured that most of the examined kinase were bound and cleaved by NS3/NS4A during the first turnover. In contrast, many reports used steady-state kinetic assays in which a small amount of NS3/NS4A was used to digest the molar excess of a protein substrate (kinase) for long times [29,41,47,56]. The steady-state kinetic parameters including *k_cat_* and *K_M_* from these assays are difficult to kinetically interpret because they are complex functions of all reactions occurring at the enzyme active site over multiple turnovers [57,58,59,60,61]. To make it worse, the fact that NS3/NS4A has a tendency to be bound and inhibited by one of its proteolytic products [62] makes the interpretation of the steady-state kinetic parameters more challenging. Under these steady-state reaction conditions, the cleavage of a protein substrate by a protease such as NS3/NS4A may appear to be slow because of product inhibition. Furthermore, the detection of interactions between the NS3 protease domain with cellular protein targets will be difficult if their interactions are transient and weak [41,47,56]. With these factors in mind, we used 10-fold more NS3/NS4A (6.4 μM) over the kinase substrate (0.6 μM) to enhance the binding interaction between the protease and its protein substrate. We were able to demonstrate that the NS3/NS4A indeed cleaved IKKα, IKKβ, IKKε, and TBK1 which were expressed and purified from baculovirus-infected insect cells (Figure 2). To further support the results from the cleavage assays, the kinases were first treated with a small amount of NS3/NS4A various times and then assayed for their remaining kinase activities (Figure 4, Figure 5, Figure 6 and Figure 7). Surprisingly, the kinase activities of IKKε and TBK1 were significantly enhanced by appropriate levels of NS3/NS4A cleavage while the opposite results were observed with IKKα and IKKβ (Figure 4, Figure 5, Figure 6 and Figure 7). In contrast to our observation, the in vitro cleavage of IKKε and TBK1 by NS3/NS4A was not observed previously by Li et al. [29]. In their digestion assays, the kinases were synthesized from in vitro translation reactions, rather than purified from baculovirus-infected insect cells, the cleavage reaction buffer was different from ours and not optimized, and the molar ratio between NS3/NS4A and each kinase was unknown. In addition to these factors, the components in Flexi-Rabbit reticulocyte lysate may inhibit the cleavage of IKKε and TBK1 by NS3/NS4A in their in vitro reactions [29].

Notably, contradicting results for the cleavage of IKKε and TBK1 by NS3/NS4A in cell-based assays can be found in the literature. In one publication, neither IKKε nor TBK1 is cleaved by NS3/NS4A when each of these kinases is individually co-transfected with NS3/NS4A into an osteosarcoma cell line UNS3-4A-24 [29]. Similarly, Breiman et al. [63] who also perform co-transfection of NS3/NS4A with each of these kinases but in a different cell line (HEK 293T cells) conclude that NS3/NS4A does not cleave IKKε and TBK1. Notably, in Figure 2B of reference [63], it is shown that in cells transfected with 5 μg IKKε DNA, the IRF-3P level (phosphorylation of IRF-3) is considerably higher in the presence than in the absence of NS3/NS4A. However, the cells that are transfected with less IKKε DNA (0.2, 1, and 2 μg) do not show any difference in the level of IRF-3 phosphorylation in the presence or absence of NS3/NS4A. To reanalyze their results based on our current knowledge, we would suggest that a higher level of IKKε expression, resulting from 5 μg IKKε DNA transfection, increases the binding interaction between IKKε and NS3/NS4A and subsequent cleavage of IKKε by the protease, leading to the enhanced phosphorylation of IRF-3 in the cells. This scenario is likely considering that the binding affinity between IKKε and the protease domain of NS3/NS4A was moderate with a *K_d_* in the low micromolar range (Figure 3).

More interestingly, our data (Figure 4, Figure 5, Figure 6 and Figure 7) indicate that NS3/NS4A mediated cleavage of cellular kinases significantly affected their kinase activities in a time-dependent manner. For example, the kinase activities of IKKα and IKKβ were reduced by NS3/NS4A treatment (Figure 4 and Figure 5) and cannot phosphorylate inhibitor IκBα, leading to a direct inhibition to NF-κB activation in vivo [32,33]. This represents another mechanism of viral immune evasion. In stark contrast, with the cleavage of IKKε and TBK1 by NS3/NS4A within the first few minutes the kinase activities of IKKε (Figure 6) and TBK1 (Figure 7) to phosphorylate IκBα were enhanced more than 10-fold. We further showed that the enhancement in kinase activity in IKKε by NS3/NS4A was dependent upon its proteolytic activity because pretreatment of NS3/NS4A with Pefabloc SC eliminated any effect of the protease on the kinase activity of IKKε (Figure 8). Pefabloc SC is a serine protease inhibitor that acylated highly conserved Ser139, one of the NS3 protease catalytic triad residues [19], leading to the inactivation of this protease activity. If occurring in vivo, the initial cleavage-caused kinase activity enhancement will facilitate the activation of both IKKε- and TBK1-dependent cellular antiviral pathways, leading to enhanced immune responses to the early stage of HCV infection. This could be one of the reasons for many patients whose acute HCV infection is controlled and rapidly cleared from the blood [64]. After NS3/NS4A cleavage for extended times, the kinase activities of IKKε (Figure 6) and TBK1 (Figure 7), just like those of IKKα (Figure 4) and IKKβ (Figure 5), were eventually eliminated. Thus, NS3/NS4A eventually allows HCV to evade cellular immune responses and establish a chronic infection.

**Figure 9:**
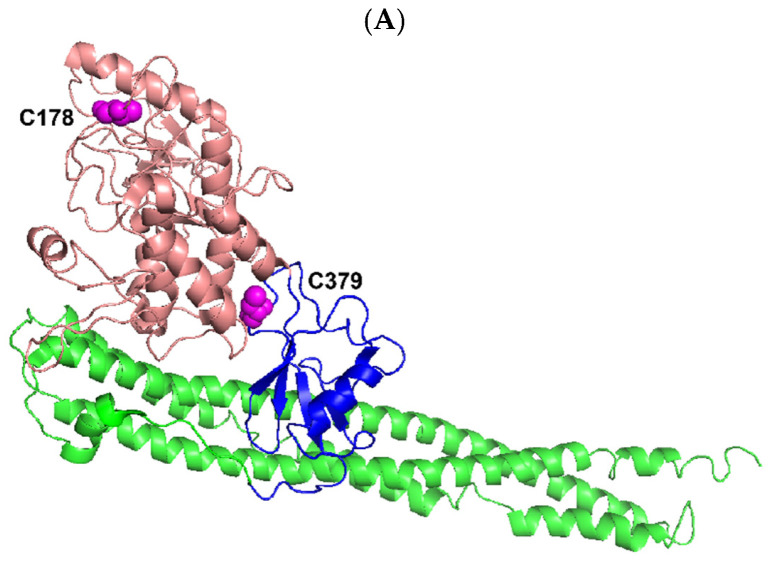

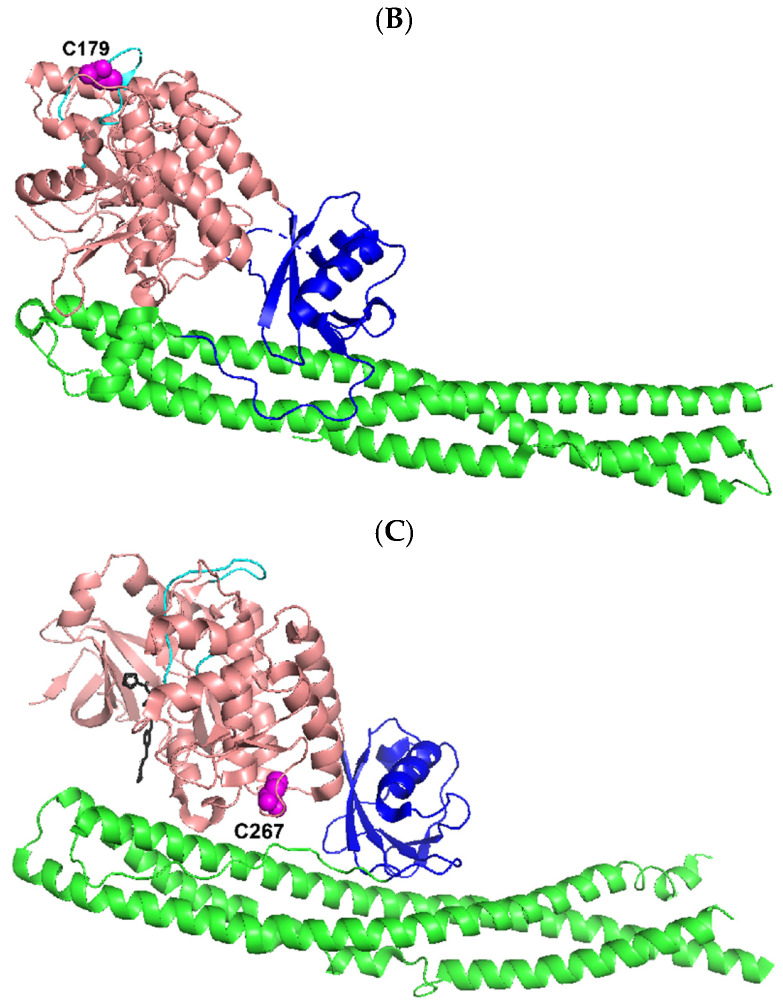
Ribbon diagram representation of the X-ray crystal structures of three truncated human IKK kinases generated by using PYMOL [68]. The N-terminal KD (salmon) including its activation loops (cyan), ULD (blue), and SDD (green) are shown while the CTD is missing. (**A**) IKKα (residues 6–660) (PDB: 5EBZ) [35]. The phosphoacceptor sites S176 and S180 of the kinase activation loop (cyan) were mutated to glutamate to mimic their phosphorylation status. HCV NS3/NS4A was predicted to cleave after C178 (magenta, spheres) within the kinase action loop and C379 (magenta, spheres) in the ULD (Table 1). (**B**) IKKβ (residues 1–671) complexed with a staurosporine analog inhibitor K252a (black) (PDB: 4KIK) [69]. The kinase activation loop (cyan) has three disordered residues (residues 174–176) and its phosphoacceptor sites (S177 and S181) were phosphorylated. NS3/NS4A was predicted to cleave after C179 (magenta, spheres) within the kinase action loop (Table 1). (**C**) TBK1 (residues 1–657) complexed with a specific inhibitor BX7 (black) (PDB: 4IW0) [38]. The kinase activation loop (cyan) was well ordered and its sole phosphoacceptor site S172 was phosphorylated. NS3/NS4A was predicted to cleave after C267 (magenta, spheres) within the KD (Table 1).

**Figure 10:**
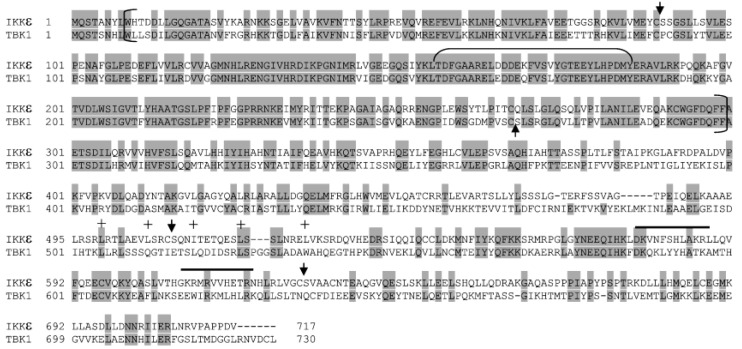
Alignment of protein sequences of human IKKε and TBK1. The solid backgrounds indicate identical amino acid residues. Vertical brackets define the KD, while the horizontal bracket denotes the kinase activation loop. The heptad repeats of hydrophobic residues within the putative leucine zipper domain are indicated with “+” symbols. Two helices of the potential helix-loop-helix domain are overlined. These domains were predicted previously by Shimada et al. [65] and Peters et al. [45] and mostly validated by the crystal structures of human TBK1 [37,38]. The predicated NS3/NS4A cleavage sites on IKKε and TBK1 (Table 1) are represented by arrowheads on top (IKKε) or bottom (TBK1) of their protein sequences.

At present, we can only speculate on how the cleavage of four canonical and noncanonical IKK kinases (IKKα, IKKβ, IKKε, and TBK1) by NS3/NS4A enhanced or abolished their kinase activities (Figure 4, Figure 5, Figure 6 and Figure 7). Human IKK kinases share 28% sequence identity and a similar domain structural organization with an N-terminal kinase domain (KD) followed by a ubiquitin-like domain (ULD), a helical scaffold dimerization domain (SDD), and a C-terminal domain (CTD) as illustrated by the solved structures of C-terminal truncated IKKα, IKKβ, and TBK1 (Figure 9) [38]. Notably, the structure of IKKε has not been made available yet. The kinase activities of IKKα and IKKβ are regulated by phosphorylation on two serine residues within their classical kinase activation loops [37]. In comparison, the phosphorylation of a single serine residue in the activation loops of IKKε and TBK1 activates the kinase activities of IKKε and TBK1 [37]. Several predicted HCV NS3/NS4A cleavage sites (Table 1) were mapped onto the structures of truncated IKKα, IKKβ, and TBK1 with activated KDs (Figure 9). Based on the KDs of IKKε and TBK1 [37,38,45,65] (Figure 9C and Figure 10), the sizes of the main cleavage products (Figure 2), and our MALDI-TOF and Nano-LC MS/MS analysis of the protein sequence of the 70 kDa cleavage product of IKKε (Figure 2), cleavage after Cys89 in IKKε and after Cys267 in TBK1 occurred within their respective KDs (see Results). The first possible scenario is that the kinase activation loop in the cleaved KD of TBK1 (or IKKε) can be more efficiently trans-autophosphorylated for its kinase activation than the same kinase activation loop in intact TBK1 (or IKKε), which is believed to be trans-autophosphorylated through higher-order oligomerization of TBK1 (or IKKε) dimers [38]. The second possible scenario is that their KDs were cleaved and reassembled due to protein-protein interaction between different kinase cleavage products that remained in the same reaction solution. Cleavage of the KDs might cause these kinases to adopt conformations that were more catalytically efficient than the intact IKKε and TBK1. The third possible scenario is that both IKKε and TBK1 possess an autoinhibitory domain. The NS3/NS4A cleavage of these autoinhibitory domains led to the up-regulation of the kinase activities of IKKε (Figure 6) and TBK1 (Figure 7). Interestingly, both N- and C-terminal autoinhibitory domains have been identified in other kinase families, and these autoinhibitory domains usually lie outside the kinase catalytic domain [66,67]. Because the likely cleavage site of TBK1 (after Cys267) by NS3/NS4A is near the C-terminus of the KD (Figure 9C and Figure 10), we speculate that the autoinhibitory domain is within the C-terminus of the KD in TBK1. In comparison, such an autoinhibitory domain was expected to lie within the N-terminal 89 amino acid residues of IKKε since this region was cleaved off IKKε by NS3/NS4A and not observed in Figure 2 (see above discussion). Future studies are needed to further investigate these three mechanistic scenarios regarding the correlation between the cleavage of IKKε and TBK1 by HCV NS3/NS4A and the kinase activities of the two noncanonical IKK kinases.

Human IKKα and IKKβ are highly homologous canonical IKK kinases [35]. Notably, HCV NS3/NS4A cleaved both IKKα and IKKβ into several fragments (Figure 2). One of the predicted cleavage sites in both IKKα and IKKβ is in their respective kinase activation loops containing the mitogen-activated protein (MAP)-kinase kinase consensus motif SXXXS, where X is any amino acid residue [70,71,72,73] (S176 and S180 in IKKα, S177 and S181 in IKKβ, Table 1). The X-ray crystal and cryo-EM structures of human IKKα [35], and the X-ray crystal structures of human IKKβ [69,74] show that their classical kinase activation loops protrude outside of their KDs before the phosphorylation of two serine residues and subsequent kinase activation, and are well exposed to solvent even after serine phosphorylation (Figure 9A,B). In other words, the kinase activation loops in IKKα and IKKβ, regardless of their serine phosphorylation status, will be accessible by HCV NS3/NS4A for cleavage. Consistently, the kinase activation loops are predicted to be cleaved by NS3/NS4A right after C178 in IKKα and C179 in IKKβ (Table 1) and these cysteine residues are on the surface of activated IKKα and IKKβ (Figure 9A,B). Thus, we hypothesize that the observed time-dependent decreases in the kinase activities of IKKα and IKKβ (Figure 4 and Figure 5) were mainly due to the gradual cleavage of the kinase activation loops in these kinases by HCV NS3/NS4A. It is possible that the kinase activity decreases also contributed through the NS3/NS4A cleavage at other predicated sites of the KDs of IKKα and IKKβ (Table 1), e.g., the cleavage of the ULD of IKKα (Figure 9A). The exact NS3/NS4A cleavage sites in IKKα and IKKβ will be unambiguously identified through future sequencing of the isolated peptide fragments by mass spectrometry analysis.

## 5. Conclusions

In this paper, we discovered that the HCV protease complex of NS3/NS4A was able to cleave human IKKα, IKKβ, IKKε, and TBK1. The cleavage of IKKα and IKKβ decreased their kinase activities in a time-dependent manner. Surprisingly, the short cleavage time of IKKε and TBK1 enhanced their phosphorylation of the protein substrate IκBα and the scenarios for such an increase in the kinase activities were discussed. After a long cleavage time by NS3/NS4A, IKKε and TBK1 were cleaved into small peptides and their kinase activities were gradually decreased and eventually lost.

## Figures and Tables

**Table 1:** Sequence alignment of known NS3/NS4A cleavage sites on HCV polyprotein precursor and putative NS3/NS4A cleavage sites on IKKα, IKKβ, IKKε, and TBK1. The conserved residues are in bold-face and the arrows depict the cleavage sites.

Proteins	Residue Number of P6	P6 P5 P4 P3 P2 P1 ↓ P1′ P2′ P3′ P4′
NS4A/NS4B		**D** E M E E **C** ↓ **A** S H L
NS4B/NS5A		**D** C S T P **C** ↓ **S** G S W
NS5A/NS5B		**E** D V V C **C** ↓ **S** M S Y
IKKα	173	**D** Q G S L **C** ↓ T S F V
	374	**D** G V R G **C** ↓ D S Y M
	691	**D** H S L S **C** ↓ V V T P
	711	**E** E N L N **C** ↓ L G H L
IKKβ	174	**D** Q G S L **C** ↓ T S F V
	711	**E** A H N L **C** ↓ T L L E
IKKε	84	L V M E Y **C** ↓ **S** S G S
	504	**E** V L S R **C** ↓ **S** Q N I
	621	L R L V G **C** ↓ **S** V A A
TBK1	262	**D** M P V S **C** ↓ **S** L S R

## Data Availability

All data generated for the current study are available from the corresponding author on reasonable request.

## Abbreviations

HCV, hepatitis C virus; IKKα, Inhibitor of κB kinase α; IκBα, Inhibitor of NF-κB α; IRF, interferon regulatory factor; MALDI-TOF, Matrix-Assisted Laser Desorption/Ionization Time-Of-Flight mass spectrometry; Nano-LC MS/MS, capillary-liquid chromatography tandem mass spectrometry; NF-κB, nuclear factor-κB; NS, nonstructural proteins; TBK1, TANK-binding kinase 1.

## References

[1] Cohen J. (1999). The Scientific Challenge of Hepatitis C. Science.

[2] Polaris Observatory H.C.V.C. (2022). Global change in hepatitis C virus prevalence and cascade of care between 2015 and 2020: A modelling study. Lancet Gastroenterol. Hepatol..

[3] Waheed Y. (2021). Progress on global hepatitis elimination targets. World J. Gastroenterol..

[4] Dhiman R.K., Premkumar M. (2020). Hepatitis C Virus Elimination by 2030: Conquering Mount Improbable. Clin. Liver Dis..

[5] Ishihama A., Nagata K. (1988). Viral RNA polymerase. Crit. Rev. Biochem..

[6] Rice C.M., Lenches E.M., Eddy S.R., Shin S.J., Sheets R.L., Strauss J.H. (1985). Nucleotide sequence of yellow fever virus: Implication for flavivirus gene expression and evolution. Science.

[7] Takamizawa A., Mori C., Fuke L., Manabe S., Murakami S., Fujita J., Onishi E., Andoh T., Yoshida I., Okayama H. (1991). Structure and organization of the hepatitis C virus genome isolated from human carriers. J. Virol..

[8] Nakano T., Lau G.M., Lau G.M., Sugiyama M., Mizokami M. (2012). An updated analysis of hepatitis C virus genotypes and subtypes based on the complete coding region. Liver Int..

[9] Grakoui A., McCourt D.W., Wychowski C., Feinstone S.M., Rice C.M. (1993). Characterization of the hepatitis C virus-encoded serine proteinase: Determination of proteinase-dependent polyprotein cleavage sites. J. Virol..

[10] Lin C., Lindenbach B.D., Prágai B.M., McCourt D.W., Rice C.M. (1994). Processing in the hepatitis C virus E2-NS2 region: Identification of p7 and two distinct E2-specific products with different termini. J. Virol..

[11] Mizushima H., Hijikata M., Asabe S., Hirota M., Kimura K., Shimotohno K. (1994). Two hepatitis C virus glycoprotein E2 products with different C termini. J. Virol..

[12] Failla C., Tomei L., De Francesco R. (1994). Both NS3 and NS4A are required for proteolytic processing of hepatitis C virus nonstructural proteins. J. Virol..

[13] Tomei L., Failla C., Santolini E., Francesco R.D., Monica N.L. (1993). NS3 is a serine protease required for processing of hepatitis C virus polyprotein. J. Virol..

[14] Bartenschlager R., Lohmann V., Wilkinson T., Koch J.O. (1995). Complex formation between the NS3 serine-type proteinase of the hepatitis C virus and NS4A and its importance for polyprotein maturation. J. Virol..

[15] Hahm B., Han D.S., Back S.H., Song O.-K., Cho M.-J., Kim C.-J., Shimotohno K., Jang S.K. (1995). NS3-4A of hepatitis C virus is a chymotrypsin-like protease. J. Virol..

[16] Jin L., Peterson D.L. (1995). Expression, isolation and characterization of the hepatitis C virus ATPase/RNA helicase. Arch. Biochem. Biophys..

[17] Kim D.W., Gwack Y., Han J.H., Choe J. (1995). C-terminal domain of the hepatitis C virus NS3 protein contains an RNA helicase activity. Biochem. Biophys. Res. Commun..

[18] Cento V., Mirabelli C., Salpini R., Dimonte S., Artese A., Costa G., Mercurio F., Svicher V., Parrotta L., Bertoli A. (2012). HCV genotypes are differently prone to the development of resistance to linear and macrocyclic protease inhibitors. PLoS ONE.

[19] Yao N., Reichert P., Taremi S.S., Prosise W.W., Weber P.C. (1999). Molecular views of viral polyprotein processing revealed by the crystal structure of the hepatitis C virus bifunctional protease-helicase. Structure.

[20] Ganta N.M., Gedda G., Rathnakar B., Satyanarayana M., Yamajala B., Ahsan M.J., Jadav S.S., Balaraju T. (2019). A review on HCV inhibitors: Significance of non-structural polyproteins. Eur. J. Med. Chem..

[21] Kish T., Aziz A., Sorio M. (2017). Hepatitis C in a New Era: A Review of Current Therapies. P T..

[22] Borowski P., Oehlmann K., Heiland M., Laufs R. (1997). Nonstructural Protein 3 of hepatitis C virus blocks the distribution of the free catalytic subunit of cyclic AMP-dependent protein kinase. J. Virol..

[23] Sakamuro D., Furukawa T., Takegami T. (1995). Hepatitis C Virus Nonstructural Protein NS3 Transforms NIH 3T3 Cells. J. Virol..

[24] Zemel R., Gerechet S., Greif H., Bachmatove L., Birk Y., Golan-Goldhirsh A., Kunin M., Berdichevsky Y., Benhar I., Tur-Kaspa R. (2001). Cell transformation induced by hepatitis C virus NS3 serine protease. J. Viral Hepat..

[25] Feng D.-Y., Sun Y., Cheng R.-X., Ouyang X.-M., Zheng H. (2005). Effect of hepatitis C virus nonstructural protein NS3 on proliferation and MAPK phosphorylation of normal hepatocyte line. World J. Gastroenterol..

[26] Dolganiuc A., Oak S., Kodys K., Golenbock D.T., Finberg R.W., Kurt-Jones E., Szabo G. (2004). Hepatitis C core and nonstructural 3 proteins trigger toll-like receptor 2-mediated pathways and inflammation activation. Gastroenterobiology.

[27] Foy E., Li K., Wang C., Sumpter R.J., Ikeda M., Lemon S.M., Gale M.J. (2003). Regulation of interferon regulatory factor-3 by the hepatitis C virus serine protease. Science.

[28] Foy E., Li K., Sumpter R., Loo Y.M., Johnson C.L., Wang C., Fish P.M., Yoneyama M., Fujita T., Lemon S.M. (2005). Control of antiviral defenses through hepatitis C virus disruption of retinoic acid-inducible gene-I signaling. Proc. Natl. Acad. Sci. USA.

[29] Li K., Foy E., Ferreon J.C., Nakamura M., Ferreon A.C.M., Ikeda M., Ray S.C., Gale M.J., Lemon S.M. (2005). Immune evasion by hepatitis C virus NS3/4A protease-mediated cleavage of the Toll-like receptor 3 adaptor protein TRIF. Proc. Natl. Acad. Sci. USA.

[30] Li X.-D., Sun L., Seth R.B., Pineda G., Chen Z.J. (2005). Hepatitis C virus protease NS3/4A cleaves mitochondrial antiviral signaling protein off the mitochondria to evade innate immunity. Proc. Natl. Acad. Sci. USA.

[31] Meylan E., Curran J., Hofmann K., Moradpur D., Binder M., Bartenschlager R., Tschopp J. (2005). Cardif is an adaptor protein in the RIG-I antiviral pathway and is targeted by hepatitis C virus. Nature.

[32] Stephenson A.A., Taggart D.J., Xu G., Fowler J.D., Wu H., Suo Z. (2022). The inhibitor of kappaB kinase beta (IKKbeta) phosphorylates IkappaBalpha twice in a single binding event through a sequential mechanism. J. Biol. Chem..

[33] Xu G., Lo Y.C., Li Q., Napolitano G., Wu X., Jiang X., Dreano M., Karin M., Wu H. (2011). Crystal structure of inhibitor of kappaB kinase beta. Nature.

[34] Xiao C., Ghosh S. (2005). NF-kappaB, an evolutionarily conserved mediator of immune and inflammatory responses. Adv. Exp. Med. Biol..

[35] Polley S., Passos D.O., Huang D.B., Mulero M.C., Mazumder A., Biswas T., Verma I.M., Lyumkis D., Ghosh G. (2016). Structural Basis for the Activation of IKK1/alpha. Cell Rep..

[36] Karin M., Ben-Neriah Y. (2000). Phosphorylation meets ubiquitination: The control of NF-[kappa]B activity. Annu. Rev. Immunol..

[37] Ma X., Helgason E., Phung Q.T., Quan C.L., Iyer R.S., Lee M.W., Bowman K.K., Starovasnik M.A., Dueber E.C. (2012). Molecular basis of Tank-binding kinase 1 activation by transautophosphorylation. Proc. Natl. Acad. Sci. USA.

[38] Larabi A., Devos J.M., Ng S.L., Nanao M.H., Round A., Maniatis T., Panne D. (2013). Crystal structure and mechanism of activation of TANK-binding kinase 1. Cell Rep..

[39] Failla C., Tomei L., De Francesco R. (1995). An amino-terminal domain of the hepatitis C virus NS3 protease is essential for interaction with NS4A. J. Virol..

[40] Zhang L., Brown J.A., Newmister S.A., Suo Z. (2009). Polymerization fidelity of a replicative DNA polymerase from the hyperthermophilic archaeon Sulfolobus solfataricus P2. Biochemistry.

[41] Steinkuhler C., Urbani A., Tomei L., Biasiol G., Sardana M., Bianchi E., Pessi A., De Francesco R. (1996). Activity of purified hepatitis C virus protease NS3 on peptide substrates. J. Virol..

[42] Wong J.H., Brown J.A., Suo Z., Blum P., Nohmi T., Ling H. (2010). Structural insight into dynamic bypass of the major cisplatin-DNA adduct by Y-family polymerase Dpo4. EMBO J..

[43] Brown J.A., Newmister S.A., Fiala K.A., Suo Z. (2008). Mechanism of double-base lesion bypass catalyzed by a Y-family DNA polymerase. Nucleic Acids Res..

[44] Lee F.S., Peters R.T., Dang L.C., Maniatis T. (1998). MEKK1 activates both IkB kinase a, IkB kinase b. Proc. Natl. Acad. Sci. USA.

[45] Peters R.T., Liao S.-M., Maniatis T. (2000). IKKe is part of a novel PMA-inducible IkB kinase complex. Mol. Cell.

[46] Bianchi E., Urbani A., Biasiol G., Brunetti M., Pessi A., Defrancesco R., Steinkuhler C. (1997). Complex formation between the hepatitis C virus serine protease and a synthetic NS4A cofactor peptide. Biochemistry.

[47] Sardana V.V., Blue J.T., Zugay-Murphy J., Sardana M.K., Kuo L.C. (1999). An uniquely purified HCV NS3 protease and NS4A(21-34) peptide form a highly active serine protease complex in peptide hydrolysis. Protein Expr. Purif..

[48] Pizzi E., Tramontano A., Tomei L., La Monica N., Failla C., Sardana M., Wood T., De Francesco R. (1994). Molecular model of the specificity pocket of the hepatitis C virus protease: Implications for substrate recognition. Proc. Natl. Acad. Sci. USA.

[49] Taliani M., Bianchi E., Narjes F., Fossatelli M., Urbani A., Steinkuhler C., De Francesco R., Pessi A. (1996). A continuous assay of hepatitis C virus protease based on resonance energy transfer depsipeptide substrates. Anal. Biochem..

[50] Fiala K.A., Suo Z. (2004). Pre-Steady-State Kinetic Studies of the Fidelity of Sulfolobus solfataricus P2 DNA Polymerase IV. Biochemistry.

[51] Sherrer S.M., Beyer D.C., Xia C.X., Fowler J.D., Suo Z. (2010). Kinetic Basis of Sugar Selection by a Y-Family DNA Polymerase from Sulfolobus solfataricus P2. Biochemistry.

[52] Maxwell B.A., Suo Z. (2012). Kinetic Basis for the Differing Response to an Oxidative Lesion by a Replicative and a Lesion Bypass DNA Polymerase from Solfolobus solfataricus. Biochemistry.

[53] Landro J.A., Raybuck S.A., Luong Y.P., O’Malley E.T., Harbeson S.L., Morgenstern K.A., Rao G., Livingston D.J. (1997). Mechanistic role of an NS4A peptide cofactor with the truncated NS3 protease of hepatitis C virus: Elucidation of the NS4A stimulatory effect via kinetic analysis and inhibitor mapping. Biochemistry.

[54] Tellinghuisen T.L., Rice C.M. (2002). Interaction between hepatitis C virus proteins and host cell factors. Curr. Opin. Microbiol..

[55] Otsuka M., Kato N., Moriyama M., Taniguchi H., Wang Y., Dharel N., Kawabe T., Omata M. (2005). Interaction Between the HCV NS3 Protein and the Host TBK1 Protein Leads to Inhibition of Cellular Antiviral Responses. Hepatology.

[56] Urbani A., Bianchi E., Narjes F., Tramontano A., De Francesco R., Steinkuhler C., Pessi A. (1997). Substrate specificity of the hepatitis C virus serine protease NS3. J. Biol. Chem..

[57] Johnson K.A., Sigman D.S. (1992). Transient-state kinetic analysis of enzyme reaction pathways. The Enzymes.

[58] Zahurancik W.J., Klein S.J., Suo Z. (2013). Kinetic Mechanism of DNA Polymerization Catalyzed by Human DNA Polymerase epsilon. Biochemistry.

[59] Fiala K.A., Suo Z. (2004). Mechanism of DNA Polymerization Catalyzed by Sulfolobus solfataricus P2 DNA Polymerase IV. Biochemistry.

[60] Brown J.A., Suo Z. (2009). Elucidating the kinetic mechanism of DNA polymerization catalyzed by Sulfolobus solfataricus P2 DNA polymerase B1. Biochemistry.

[61] Kumar A., Reed A.J., Zahurancik W.J., Daskalova S.M., Hecht S.M., Suo Z. (2022). Interlocking activities of DNA polymerase beta in the base excision repair pathway. Proc. Natl. Acad. Sci. USA.

[62] Steinkuhler C., Biasiol G., Brunetti M., Urbani A., Koch U., Cortese R., Pessi A., De Francesco R. (1998). Product inhibition of the hepatitis C virus NS3 protease. Biochemistry.

[63] Breiman A., Grandvaux N., Lin R., Ottone C., Akira S., Yoneyama M., Fujita T., Hiscott J., Meurs E.F. (2005). Inhibition of RIG-I-dependent signaling to the interferon pathway during hepatitis C virus expression and restoration of signaling by IKKepsilon. J. Virol..

[64] Hoofnagle J.H. (1999). Management of hepatitis C: Current and future perspective. J. Hepatol..

[65] Shimada T., Kawai T., Takeda K., Matsumoto M., Inoue J., Tatsumi Y., Kanamaru A., Akira S. (1999). IKK-i, a novel lipopolysaccharide-inducible kinase that is related to IkappaB kinases. Int. Immunol..

[66] Cohen L.A., Guan J.-L. (2005). Residues within the first subdomain of the FERM-like domain in Focal Adhesion Kinase are important in its regulation. J. Biol. Chem..

[67] Zu Y.L., Ai Y., Huang C.K. (1995). Characterization of an autoinhibitory domain in human mitogen-activated protein kinase-activated protein kinase 2. J. Biol. Chem..

[68] DeLano W.L. (2002). The PyMOL Molecular Graphics System.

[69] Liu S., Misquitta Y.R., Olland A., Johnson M.A., Kelleher K.S., Kriz R., Lin L.L., Stahl M., Mosyak L. (2013). Crystal structure of a human IkappaB kinase beta asymmetric dimer. J. Biol. Chem..

[70] Woronicz J.D., Gao X., Cao Z., Rothe M., Goeddel D.V. (1997). IkappaB kinase-beta: NF-kappaB activation and complex formation with IkappaB kinase-alpha and NIK. Science.

[71] Zandi E., Rothwarf D.M., Delhase M., Hayakawa M., Karin M. (1997). The IkappaB kinase complex (IKK) contains two kinase subunits, IKKalpha and IKKbeta, necessary for IkappaB phosphorylation and NF-kappaB activation. Cell.

[72] Mercurio F., Zhu H., Murray B.W., Shevchenko A., Bennett B.L., Li J., Young D.B., Barbosa M., Mann M., Manning A. (1997). IKK-1 and IKK-2: Cytokine-activated IkappaB kinases essential for NF-kappaB activation. Science.

[73] DiDonato J.A., Hayakawa M., Rothwarf D.M., Zandi E., Karin M. (1997). A cytokine-responsive IkappaB kinase that activates the transcription factor NF-kappaB. Nature.

[74] Polley S., Huang D.B., Hauenstein A.V., Fusco A.J., Zhong X., Vu D., Schrofelbauer B., Kim Y., Hoffmann A., Verma I.M. (2013). A structural basis for IkappaB kinase 2 activation via oligomerization-dependent trans auto-phosphorylation. PLoS Biol..

